# Cholecystokinin/sulfakinin peptide signaling: conserved roles at the intersection between feeding, mating and aggression

**DOI:** 10.1007/s00018-022-04214-4

**Published:** 2022-03-14

**Authors:** Dick R. Nässel, Shun-Fan Wu

**Affiliations:** 1 Department of Zoology, Stockholm University, 10691 Stockholm, Sweden; 2 College of Plant Protection/Laboratory of Bio-Interactions and Crop Health, Nanjing Agricultural University, Nanjing, 210095 China

**Keywords:** Sulfakinin, *Drosophila*, Neuromodulation, Peptide hormone, Satiety, Behavior

## Abstract

Neuropeptides are the most diverse messenger molecules in metazoans and are involved in regulation of daily physiology and a wide array of behaviors. Some neuropeptides and their cognate receptors are structurally and functionally well conserved over evolution in bilaterian animals. Among these are peptides related to gastrin and cholecystokinin (CCK). In mammals, CCK is produced by intestinal endocrine cells and brain neurons, and regulates gall bladder contractions, pancreatic enzyme secretion, gut functions, satiety and food intake. Additionally, CCK plays important roles in neuromodulation in several brain circuits that regulate reward, anxiety, aggression and sexual behavior. In invertebrates, CCK-type peptides (sulfakinins, SKs) are, with a few exceptions, produced by brain neurons only. Common among invertebrates is that SKs mediate satiety and regulate food ingestion by a variety of mechanisms. Also regulation of secretion of digestive enzymes has been reported. Studies of the genetically tractable fly *Drosophila* have advanced our understanding of SK signaling mechanisms in regulation of satiety and feeding, but also in gustatory sensitivity, locomotor activity, aggression and reproductive behavior. A set of eight SK-expressing brain neurons plays important roles in regulation of these competing behaviors. In males, they integrate internal state and external stimuli to diminish sex drive and increase aggression. The same neurons also diminish sugar gustation, induce satiety and reduce feeding. Although several functional roles of CCK/SK signaling appear conserved between *Drosophila* and mammals, available data suggest that the underlying mechanisms differ.

**Supplementary Information:**

The online version contains supplementary material available at 10.1007/s00018-022-04214-4.

## Introduction

Neuropeptides are involved in the regulation of physiology and a wide array of vital behaviors of metazoans. They constitute secreted signals in neuronal circuits that are hierarchically arranged in the brain and partake in context-dependent orchestrating signaling by higher-order neurons, as well as in local executive modulation in specific circuits (see [1–5]). Thus, neuropeptides impart plasticity to the hardwired circuits of the central nervous system and in this review we highlight some specific roles of neuropeptides, especially cholecystokinin-like peptides, in regulation of competing behaviors such as feeding, mating and aggression.

Neuropeptides and peptide hormones constitute ancient signaling molecules that are genetically encoded on precursors that give rise to one or more bioactive peptides acting on different types of membrane receptors. Bioactive peptides are present already in organisms that lack a nervous system, such as for instance sponges [6] and the Placozoan *Trichoplax adhaerens*, [7]. The latter small marine animal utilizes a small number of peptides, derived from five precursors, to regulate simple behaviors associated with locomotion and food intake [8]. In organisms with simple nervous systems, such as cnidarians and ctenophores, there are more diverse sets on peptides produced by neurons, and thus referred to as neuropeptides [9–12]. The more evolved animals among the Bilateria produce numerous neuropeptides as well as peptide hormones that display a wide array of diverse functions in development, physiology and behavior [13–17]. Many of these peptidergic signaling pathways are evolutionarily conserved in the bilaterian phyla, including mammals [13, 14]. Among the conserved signaling pathways are those that utilize cholecystokinin (CCK)-related peptides and their receptors. It should be noted that in mammals CCK exists alongside a closely related (paralog) peptide named gastrin, which is encoded on a separate gene. We will discuss these two peptides in Sect. 2, but in the following we will refer to CCK signaling for simplicity. Interestingly, CCK signaling is functionally pleiotropic in most animals studied and regulates behavior and physiology associated with feeding, digestion, aggression and reproduction (reviewed in [16, 18–23]).

CCK signaling components have been identified in vertebrates and in several bilaterian invertebrate phyla [13–15, 24–31], as well as in deuterostome invertebrates such as echinoderms and invertebrate chordates (the latter also known as protochordates) [32, 33]. In mammals, CCK activity was first discovered in tissue already in 1906 [34] and CCK was isolated as a gut hormone in 1928 [35]. The peptide was initially identified as a factor released from the small intestine of cats and dogs that induced contractions of the gall bladder [35], and from pig intestine that triggered secretion of pancreatic enzyme [36]. Over the years, multiple additional functions have been discovered, including regulation of satiety and various neuromodulatory roles in the brain (see [18, 19, 37]).

**Fig. 1:**
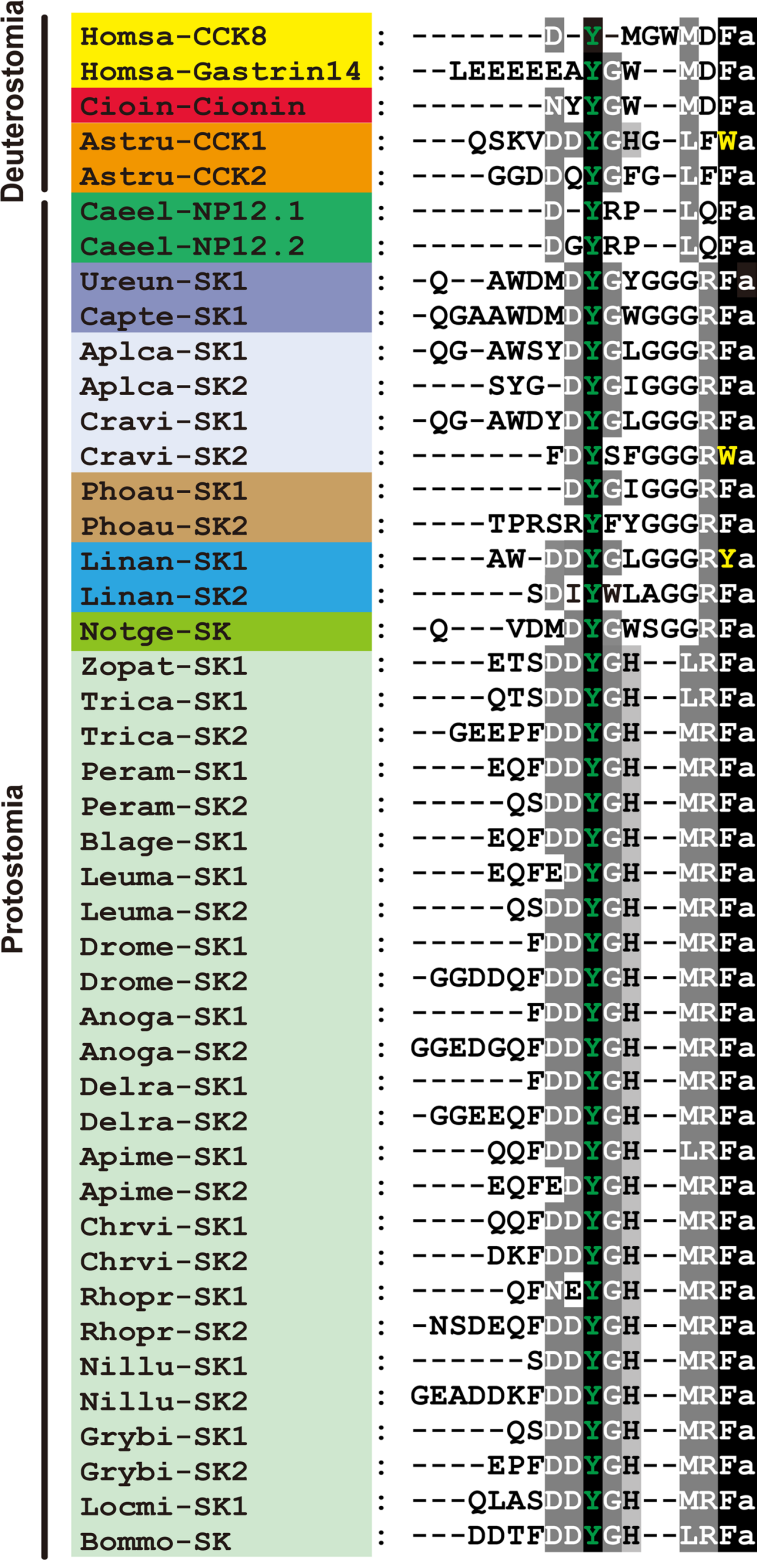
Sequence alignments of CCK, gastrin, sulfakinin and sulfakinin-like peptides from select species. Conserved residues are highlighted in black (identical) or gray (similar). Sulfated tyrosine was highlighted in green with black background. Species belonging to the same phyla have been highlighted with the same color. Note that pyroglutamate-blocked N-terminal residues are not indicated. Species names are as follows: Homsa (*Homo sapiens*), Cioin (*Ciona intestinalis*), Astru (*Asterias rubens*), Caeel (*Caenorhabditis elegans*), Ureun (*Urechis unicinctus*), Capte (*Capitella teleta*), Aplca (*Aplysia californica*), Cravi (*Crassostrea virginica*), Phoau (*Phoronis australis*), Linan (*Lingula anatina*), Notgen (*Notospermus geniculatus*), Zopat (*Zophobas atratus*), Trica (*Tribolium castaneum*), Peram (*Periplaneta americana*), Blage (*Blattella germanica*), Leuma (*Leucophaea maderae*), Drome (*Drosophila melanogaster*), Anoga (*Anopheles gambiae*), Delra (*Delia radicum*), Apime (*Apis mellifera*), Chrvi (*Chrysis viridula*), Rhopr (*Rhodnius prolixus*), Nillu (*Nilaparvata lugens*), Grybi (*Gryllus bimaculatus*), Locmi (*Locusta migratoria*), Bommo (*Bombyx mori*). Note that some insects only have one form of SK. The accession numbers of the sequences are listed in “Fig. 1 source data” in **Supplementary data files**

The presence of CCK like peptides in insects and some other invertebrates was suggested early on [38–41], but was based solely on immunochemical detection with heterologous antisera. The first invertebrate CCK-type peptide was isolated in 1986 from the cockroach *Leucophaea maderae* (now *Rhyparobia maderae*) using head extract and monitoring myostimulatory action on the hindgut [24]. This peptide was named leucosulfakinin and closely related peptides, sulfakinins (SKs), have since been identified in multiple insects and other invertebrates, as well as in invertebrate chordates (see examples in Fig. 1). However, CCK-type peptides have not been found in non-bilaterians such as Porifera, Placozoa, Cnidaria, or Ctenophora (see [13–15]). After the identification of two SK receptors in *Drosophila* [42, 43], numerous invertebrate SK receptors are now known that display homologies to those of gastrin and CCK receptors in vertebrates (see [20, 32]). Signaling with CCK-type peptides has been assayed in multiple invertebrates and in this review we highlight the structure, distribution and functions of these peptides and their receptors, with some more detailed analysis in *Drosophila* and with comparisons to mammals. More specifically, we discuss the roles of SKs in satiety signaling, feeding, metabolism, reproductive behavior and aggression. Interestingly, it has been shown that DSK neurons are important in the regulation of competing behaviors. Thus, in male flies, DSK signaling diminishes sex drive, but increases aggression, in addition to its known role in inducing satiety and reduced feeding [23, 44, 45]. This peptidergic regulation of competing behaviors is the topic of one of the sections in this review. We also discuss how CCK-mediated satiety signaling in mammals differs mechanistically from SK signaling in *Drosophila,* although the outcome on food intake is similar. Finally, we outline some other neuromodulatory systems that use neuropeptides and monoamines to regulate feeding, reproductive behavior and aggression in association with SKs.

## CCK signaling in mammals: a brief overview

Since there is a tremendous amount of published data on the distribution and functional roles of CCK and its receptors in mammals we will mainly focus on some of those features that relate to SK functions in invertebrates: satiety and feeding behavior, reproductive behavior, and aggression. For further details, several reviews are available that cover CCK signaling in mammals and other vertebrates [18, 19, 46–49].

**Fig. 2:**
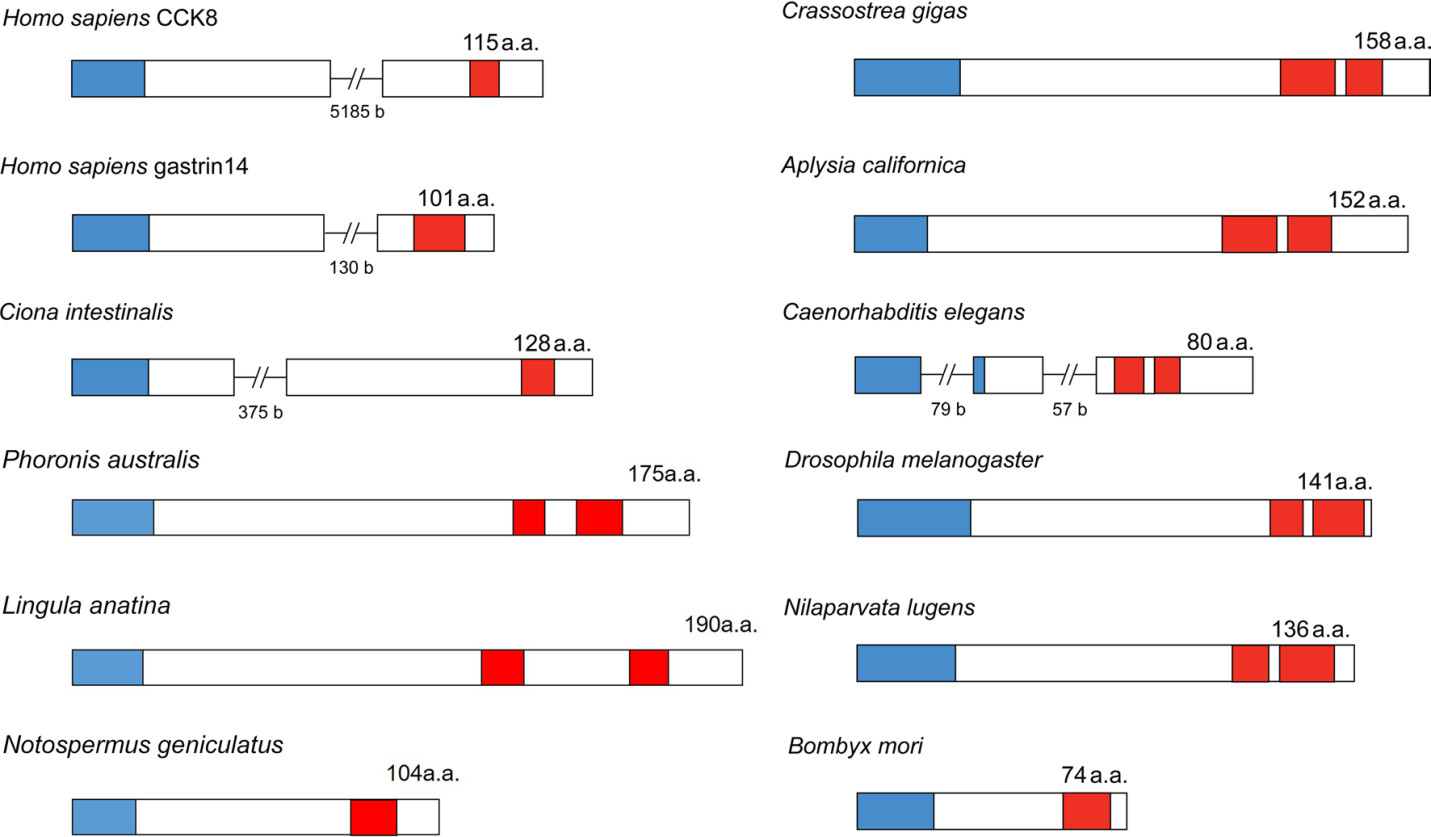
Schemes of CCK/SK precursors from representative species. Boxes and lines show exons and introns, respectively. Red boxes represent SK/CCK peptides and blue boxes indicate signal peptides. Note that DSK0 is not indicated in *Drosophila*. *Crassostrea* and *Aplysia* are mollusks, *Ciona* an invertebrate chordate, *Caenorhabditis* a nematode worm, *Phoronis australis* a phoronid, *Lingula anatine* a brachiopod, *Notospermus geniculatus* a nemertin and the others are insects. Introns have only been identified in mammals and *C. elegans.* The accession numbers of the sequences are listed in “Fig. 2 source data” in **Supplementary data files**

CCK was one of the first hormones to be discovered in mammals, and was originally isolated from the small intestine as a factor that regulates gallbladder emptying and enzyme secretion from the pancreas [34, 35]. It can be noted that before sequencing of the factors acting on gall bladder and pancreas they were named CCK [35] and pancreozymin [36], respectively. After purification and sequencing in 1968, it turned out that the two activities were derived from a single peptide CCK/pancreozymin [50] and the name pancreozymin was abandoned. CCKs display strong sequence similarities to the peptide hormone gastrin, which was actually sequenced prior to CCK [51]. Gastrin was found in the duodenum and stomach and regulates gastric acid secretion [51]. Both CCK and gastrin exist in several forms that are N-terminal extensions of the same core peptides (CCK: 8, 33, 39, 58 or 83 amino acids; gastrin: 13, 17 or 34 amino acids) (see [18, 19, 52]). Note that CCK and gastrin are encoded on separate genes/precursors (see Fig. 2). The sequence of CCK8 in mammals is DYMGWMDFamide, where the tyrosine (Y) residue is commonly sulfated. The gastrin and CCK peptides share the C-terminal sequence GWMDFamide (see Fig. 1). It is interesting to note that, like several other neuropeptides, CCK-type peptides (e.g., cerulein) have also been identified in frog skin, where they are likely to be secreted as a deterrent against predators [53].

Mammalian CCK and gastrin act on the two GPCRs designated CCK1R and CCK2R, respectively (see [49]), the first of which was cloned in 1992 [54]. These receptors have different affinities for the gastrin/CCK peptide isoforms. Thus, CCK1R has a high affinity for sulfated CCK8-CCK58 and a 1000-fold lower affinity for gastrin, whereas CCK2R can be activated by both gastrin and CCK, even in their non-sulfated forms [19, 55]. The CCK1R is distributed in peripheral tissues such as the gastrointestinal tract, gallbladder, pancreas, and also in the anterior pituitary, nucleus accumbens, posterior hypothalamus, the brainstem (notably the nucleus of the solitary tract) and some areas of the midbrain, whereas the CCK2R is distributed predominantly in the brain, including amygdala, habenula, thalamus, cortex, hippocampus, and the olfactory bulb, and also in the pancreas and some parts of the gastrointestinal tract (reviewed in [18, 19]).

CCK, especially CCK8, is distributed widely in neurons in different parts of the brain, corresponding to the sites where the two CCK receptors have been found [18, 19, 56]. Importantly, CCK is also produced by a specific type of enteroendocrine cells (EECs), classified as type I cells, present in the duodenum and jejunum of the gastrointestinal tract (see [18, 46, 48, 57, 58]). It is CCK from these EECs that induces satiety.

**Fig. 3:**
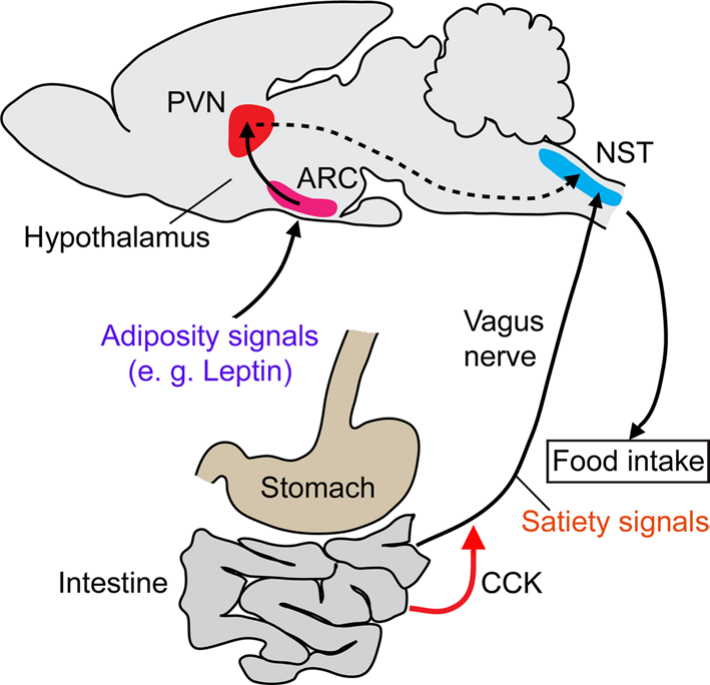
CCK and regulation of satiety in mammals. Upon stomach distension, CCK is released from enteroendocrine cells of the intestine and acts on CCK receptors on afferent neurons in the vagus nerve. These afferents signal to the nucleus of the solitary tract (NST) in the brainstem. Efferent neurons in the NST signal to regulate food intake by stomach emptying. Signals from adipose tissue (e.g., leptin) reach the neurons in the arcuate nucleus (ARC) in the hypothalamus, which in turn activate neurons in the paraventricular nucleus (PVN) and triggers signals to the NST that also regulate food ingestion. The vagus nerve afferents relay complex satiety signals that are gated by CCK (see text for further details). There is also CCK8-mediated signaling from nutrient-responsive CCK-producing neurons of the NST that innervate the PVN (not shown here). This figure is based on, but redrawn from, Morton et al. [286] and Dockray [47]

Satiety in mammals is primarily induced by CCK signaling from the gut to the brain via the vagus nerve, and the mechanisms described next are based on several reviews [18, 46–48, 55]. Upon food intake and ensuing gastric distension, CCK is released from the EECs to act on afferent neurons in the adjacent vagal nerve (Fig. 3). These vagal afferent neurons (VANs) express CCKR1 and their axons connect to neurons in the nucleus of the solitary tract of the brainstem (hindbrain) (Fig. 3). CCK binding to the CCK1R in neurons of the VANs leads to activation of these neurons and thereby triggers a post-ingestive feedback to the hindbrain. The sensitivity of the VANs to CCK is modulated by the metabolic/energy state and CCK signaling regulates the expression of genes encoding other anorectic and orexigenic peptides and receptors in the VANs (e.g., receptors for leptin, urocortin, insulin, α-MSH, ghrelin and endocannabinoids). Thus, the VANs integrate several peptidergic signals and some sensory inputs to fine-tune food intake. It has been suggested that the VANs can adopt two states depending on the expression of these peptides and receptors, one associated with feeding (hunger) and another with inhibition of feeding (satiety) and that CCK acts as a gatekeeper that determines these states (see [47]). The CCK-induced signals in VANs propagate to the brainstem and result in efferent signals to the gastrointestinal tract that reduces food intake by controlling meal size. Mechanistically, these efferent satiety signals regulate (inhibit) gastric emptying, which in turn leads to reduced food ingestion (see [18, 47]). Furthermore, the VAN signals to the nucleus of solitary tract (NST) in the brainstem lead to activation of second-order neurons expressing for example neuropeptide Y, proopiomelanocortin (POMC) and dopamine. These NST neurons innervate several brain centers that regulate reward and food ingestion, such as the hypothalamus, mesolimbic system and nigro-striatal pathway. Thus, CCK-mediated satiety signaling originating in the intestine not only activates a direct feedback to the gastrointestinal tract, but also indirectly activates signaling within the brain that may mediate more long-lasting effects on behavior.

A direct central action of CCK in satiety has also been suggested. It was for instance shown that microinjection of CCK into the dorsomedial hypothalamus in rats leads to a reduced food intake and is mediated by the CCKR2 [59]. In mice, the neuronal substrate for this CCK8-mediated satiety signaling includes nutrient-responsive CCK-producing neurons of the NST that innervate the paraventricular nucleus of the hypothalamus [60]. Activation of these CCK neurons generates a long-term effect on appetite and reduction of body weight. In contrast, the CCK feedback action via the VAN/brainstem is associated with a short-term effect on food intake.

Further roles of CCK associated with feeding and metabolism include local actions in the intestine to decrease gastrointestinal motility, stimulate secretion of pepsinogen, inhibit gastric acid secretion, stimulate gallbladder contraction, and trigger secretion of hormones in the endocrine pancreas (reviewed in [18, 19, 61]). CCK peptides also stimulate secretion of calcitonin, insulin, and glucagon that are important regulators of metabolic homeostasis (see [19]).

Some evidence for a role of CCK in reproductive behavior is available. There is a sexual difference in neuronal CCK distribution in the bed nucleus of the stria terminalis and in the amygdala in rats [21]. This sexually dimorphic distribution of CCK neurons is in regions that are targets of steroid sex hormones and that are known to regulate aspects of reproduction [21]. This suggests that CCK is involved in a central integration of sensory input and steroid hormone action to modulate reproductive behavior [21]. Furthermore, it was reported that intraperitoneal administration of CCK-8 inhibits lordosis behavior (a mating response) in female rats, but not in males [62, 63].

A role of CCK in aggression in rats and mice has also been suggested. CCK-expressing neuronal projections have been identified within the limbic system, the brainstem, and the cerebral cortex, areas known to overlap with neuronal pathways that are involved in the modulation of fear, anxiety, and aggression (see [64]). It was shown that CCK signaling through the CCK1R in the brain induces hyperactivity and aggression [65]. Furthermore, overexpression of CCK2R in the mouse brain increases aggressive behavior, whereas mice lacking CCK2R display increased exploratory behavior and reduced anxiety [65, 66]. Other-CCK mediated regulatory functions in the brain have been discovered: in sleep, nociception, memory and learning processes, panic and anxiety (see [46, 67, 68]).

## CCK/sulfakinin signaling components in invertebrates: a general overview

We now turn to signaling with CCK-type peptides, or SKs, in various invertebrate species. In this section, we describe general features of SKs, the structures of their precursors and SK receptors. We also point out structural similarities to vertebrate CCK and gastrin signaling components and evolutionary aspects of this signaling system. Finally, we discuss the distribution of peptides and receptors and their functional roles in invertebrates. A more detailed description of SK (DSK) signaling in *Drosophila* is given in Sect. 4, where we also highlight how DSK signaling serves to modulate competing behaviors.

### Structure of invertebrate SK precursors and peptides

The first invertebrate CCK-type peptide was isolated from cockroach head extract and has the sequence EQFEDYGHMRFamide, which is highly similar to mammalian gastrin and CCK (see Fig. 1), including a sulfated tyrosine [24]. This peptide was designated leucosulfakinin (LSK). A second, pyroglutamate blocked, LSK (LSK-II, pQSDDYGHMRFamide) was identified soon after [69]. The first invertebrate gene encoding a SK precursor was cloned in *Drosophila* in 1988, and can give rise to the peptides DSK1, DSK2 and DSK0 [70] and later two DSK receptors (CCKLR1 and CCKLR2) were identified and characterized [42, 43].

Now CCK-type peptides have been identified in numerous invertebrate species of several bilaterian phyla, including the protostome phyla nematodes, mollusks, nemerteans, brachiopods, phoronids, annelids, tardigrades and arthropods [13–15, 24–31, 71–73], as well as in deuterostome invertebrates such as echinoderms and invertebrate chordates [32, 33]. Examples of SK sequences from different taxa are shown in Fig. 1. In all studied invertebrates, with the exception of spiders, there is only one gene encoding SK precursors [74]. In spiders, but not scorpions and mites, there are two genes encoding SK precursors [74, 75]. One of these spider precursors encodes two SK paracopies, and the other just a single SK. The organization of select CCK/SK precursors is shown in Fig. 2.

Interestingly, it seems that the presence of SKs is variable among species of certain taxa. For instance, among arthropods, SKs have not been identified in quite a number of studied beetles [76], and are lacking in the genomes of some parasite wasps [77, 78], in pea aphids [79, 80], in a stick insect, *Carausius morosus* [81], the Asian citrus phyllid *Diaphorina citri* (hemiptera) [82] and in a spider mite [83]. Interestingly, however, another mite, the house dust mite, does produce SKs [74]. It should be mentioned here that in cases where it has been investigated the lack of SK peptides is correlated with a lack of cognate CCK-type receptor. An exception to this is seen among the Xenacoelomorphs, a group that may have branched off from the other bilaterian phyla (Nephrozoa) early in evolution or constitute a sister group of Ambulacraria (see [84]). In this group SKs have not been identified, but two clades, Xenoturbella and Nemertodermatida, possess SK-type receptors, whereas the Acoela do not [29].

An important question to ask is whether other neuropeptides functionally replace SKs in those species that lack them, or whether the functional roles played by SKs are simply missing. Since SKs seem to play major roles in satiety signaling and regulation of feeding and digestion one might suspect that lack of SKs could reflect specific feeding habits. This seems not to be the case at least in beetles (coleopterans) where 31 species were analyzed of which 13 lack SKs [76]. When comparing the presence of SKs to life history parameters, including dietary behavior (herbivorous, mixed, predacious, saprophagous and xylophagous), no obvious correlation was found [76]. In the same study the authors also found no correlation between lack of SK (and some other neuropeptides) and other life history parameters or taxonomic relatedness of species. A few other neuropeptides are also lacking in several arthropod species, such as for example adipokinetic hormone/corazonin-related peptide (ACP), allatotropin, corazonin and leucokinin (see [16, 76, 82, 83, 85]). Thus, it remains to be understood why these losses have occurred and what the functional consequences are.

There are SK sequence entries for 116 insect species in the DINeR insect neuropeptide database (http://www.neurostresspep.eu/diner [86]). These entries reveal that insects commonly have two SKs, although several have only one form (e.g., *Locusta migratoria, Bombyx mori* and *Manduca sexta*), whereas the giant springtail *Tetrodontophora bielanensis* has three (see [87]). Also the shrimp *Penaeus monodon* has three bona fide SKs [88]. In *Drosophila, melanogaster* and other *Drosophila* species the precursor encodes a third shorter peptide (DSK0), whose sequence barely resembles a bona fide DSK ([70], DINeR database) and the presence of processed DSK0 in tissue has not been confirmed by mass spectrometry (see [89]). As mentioned, SKs are missing in some insect taxa, but since already the most basal apterygote insects possess SKs [87], it is suggestive that lack of SKs in various species is the result of a secondary loss.

Also in invertebrates in general, the precursors give rise to two SK paracopies (isoforms) in each species (see Fig. 2). Often, like in the starfish *Asterias rubens* [32] and several insect species such as for instance the cockroach *P. americana* [90] and bed bug *Cimex lectularius* [91], two SKs can be identified on the precursor, but mass spectrometry reveals that these exist both in sulfated and non-sulfated forms, suggesting the presence of four structural isoforms. A recent study of the locust *Schistocerca gregaria* found that the two paracopies SK1 (12 residues) and SK2 (27 residues) can be identified by mass spectrometry of dissected tissues in three forms each: SK1 as sulfated with N-terminal pQ (pyroglutamate), nonsulfated with pQ and sulfated without pQ, and SK2 as sulfated with pQ, nonsulfated with pQ and a truncated sulfated form (9 residues) [92]. In *C. elegans* the NPL-12a and 12b peptides only display weak sequence similarities to CCK (see Fig. 1), but activate two GPCRs related to CCK receptors (T23B3.4 and Y39A3B.5) [25, 93], suggesting the presence of CCK-type signaling in this worm.

In insects the SKs are C-terminally amidated and commonly between 9 and 17 amino acids long, and in many cases a species has one short and one longer form (see DINeR Database). However, with the exception of *S. gregaria* SK2 with 27 residues, there seems not to be any drastically extended SK forms in invertebrates, in contrast to mammalian CCK33, CCK58 and gastrin34. As noted above, some of the SKs are blocked with an N-terminal pyroglutamate (pQ), which adds a resistance to certain peptidases (e.g., in *L. maderae, A. rubens, S. gregaria* and *Tribolium castaneum* [32, 69, 92, 94]). The SKs have a conserved tyrosine (Y) residue that needs to be sulfated for full bioactivity of the peptide [90, 95, 96]. Curiously, in a bivalve mollusk, *Crassostrea gigas*, in the white shrimp *Litopenaeus vannamei* and the invertebrate chordate *Ciona intestinalis,* one of the SKs was identified with two sulfated tyrosines [33, 71, 97]. Several investigations of insects have found biological activity also for non-sulfated SKs (see e.g., [98–101]). It should be noted that this activity was detected in assays different from the ones used in canonical SK signaling in insects (see Table 1) and it is not known whether these SKs act on the bona fide SK receptors. In summary, the active core sequence in insect SKs for canonical bioactivity is Y(S0_3_H)GHMRFamide [95, 96], which is well conserved also among other arthropods. As seen in Fig. 1, the sequences of other invertebrate SKs vary somewhat.

**Table 1:** Functions of sulfakinins in invertebrates

Species	Cells/neurons^a^	Function^b^		Reference
**Insects**				
*Drosophila*^*c*^	IPCs or all DSK neurons		Decreases food intake, starvation resistance and *dilp2* expression	[121]
*Drosophila*^*c*^	IPCs or all DSK neurons		Decreases food intake, increases male aggression	[140]
*Drosophila*^*c*^	All DSK neurons CCKLR-17D1		Increases male aggression, promotes social dominance	[44]
*Drosophila*^*c*^	IPCs or all DSK neurons		Increases male aggression (induced by social isolation)	[141]
*Drosophila*^*c*^	MP1/MP3 neurons CCKLR-17D3		Suppresses male sexual arousal	[23]
*Drosophila*^*c*^	MP1/MP3 neurons		Suppresses sugar gustation and mediates satiety	[45]
*Drosophila*	Peptide injections		Larval odor preference and locomotion	[98]
*Nilaparvata lugens*	Peptide injection SK knockdown^d^		Suppresses sugar gustation and mediates satiety	[45]
*Nilaparvata lugens*	Peptide injection		Reduces digestive enzyme activity	[142]
*Tribolium castaneum*	Peptide injection and SKR2 knockdown^d^		Decreases food intake	[104, 105, 113, 143]
*Schistocerca gregaria*	Peptide injection		Decreases food intake	[144]
*Gryllus bimaculatus*	SK knockdown^d^		Decreases food intake	[145]
*Blattella germanica*	Peptide injection		Decreases food intake	[146]
*Rhodnius prolixus*	Peptide injection		Decreases food intake	[116]
*Rhodnius prolixus*	SK and SKR knockdown^d^		Decreases food intake	[109]
*Phormia regina*	Peptide injection		Decreases carbohydrate intake	[147]
*Periplaneta americana*	Electrophysiology and SK application		Inhibits DUM neurons antago-nistically to AKH^e^	[118]
*Locusta migratoria*	Peptide injection		Inhibits digestive enzyme secretion	[135]
*Rhynchophorus ferrugineus*	Peptide injection		Stimulates release of α-amylase	[136]
*Leucophaea maderae*	In vitro assay		Myostimulatory on hindgut	[24]
*Zophobas atratus*	Peptide injection		Fatty acid composition and ILP levels in oenocytes,	[137]
*Tenebrio molitor*	Peptide injection		Carbohydrate and ILP levels in hemolymph of larvae	[100]
Other invertebrates				
*Homarus americanus* (lobster)	In vitro assay	Stimulates heart contractions		[148]
*Asterias rubens* (starfish)	Peptide injection	Inhibits feeding and triggers stomach retraction		[32]
*Crassostrea gigas* (oyster)	In vitro assay	Inhibits contractions in hindgut^f^		[71]
*Pecten maximus* (scallop)	Peptide injection	Stimulates release of α-amylase		[136]
*C. elegans*	Genetic manipulation	Modulates food-related behavior		[27]

^**a**^Cells/neurons where SK signaling was manipulated. In other cases global action was assayed by various techniques

^**b**^Function shows result of increased SK signaling

^*c*^Function in *Drosophila* assessed by various genetic manipulations

^d^Injection of dsRNA

^e^Inhibits octopaminergic dorsal unpaired median (DUM) neurons that regulate foraging activity antagonistically to the orexigenic peptide AKH

^f^Also suggested that SK regulates feeding

Typically, only one gene encoding a CCK/gastrin type precursor has been found in each species of protostome invertebrates [20], as well as in the deuterostome invertebrates of the Ambulacraria (echinoderms and hemichordates) [32] and in invertebrate chordates, like the ascidian *Ciona intestinalis* [102]. Thus, a duplication of an ancestral CCK/gastrin gene that gave rise to separate genes encoding CCK and gastrin in the vertebrates seem to have occurred before the emergence of cartilaginous fish [52]. More specifically, synteny analysis has shown that this duplication arose through a whole genome duplication event (the second one known, 2R), probably before the emergence of cyclostomes [103].

### Invertebrate SK receptors

Two DSK receptors (CCKLR1 and CCKLR2) were identified and characterized in *Drosophila* [42, 43] and later orthologs were characterized in *Tribolium castaneum* [104, 105]. These were followed by detection of numerous SK receptors (SKRs) in other invertebrates (see [20, 106]). Thus SKRs have been identified in several insects, as well as in for instance *C. elegans*, the water flea *Daphnia pulex*, a spiny lobster *Panulirus argus*, the sea squirt *Ciona intestinalis*, as well as the echinoderms *Strongylocentrotus purpuratus* and *Asterias rubens* (see [20, 25, 102, 107, 108]). However, cloning and characterization of SKRs has been performed only for a more limited number of species: *Drosophila, T. castaneum*, *Rhodnius prolixus, A. rubens, C. elegans* and *C. intestinalis* [20, 25, 32, 102, 105, 109]. Like in mammals and *Drosophila*, several insects and other invertebrates have two CCK-type receptors, or SKRs. However, in some only one SKR could be found in the genome: for instance brown planthopper *Nilaparvata lugens*, silkworm *Bombyx mori* and American cockroach *P. americana*, (see [20]). Also the starfish *A. rubens* seems to have only one SKR [32]. In cases that have been investigated, it appears that in insects that lack SK peptides also no SKR can be found in the genome, for example in pea aphid *Acyrthosiphon pisum*, the parasitic wasp *Nasonia vitripennis* and the parasitic nematode *Meloidogyne incognita* [20].

In *Drosophila*, sulfated DSK-1 and DSK-2 can both activate the two receptors CCKLR1 (CCKLR-17D3; CG32540) [42] and CCKLR2 (CCKLR-17D1; CG42301) [43] in different in vitro expression/assay systems. However, DSK-0, and the non-sulfated DSK-1 and DSK-2 cannot active the receptors at physiological concentrations. The signaling downstream of the CCKLRs in *Drosophila* has been analyzed to some extent. DSK/CCKLR2 regulation of larval neuromuscular junction growth was found to be mediated by the cyclic adenosine monophosphate (cAMP)—protein kinase A (PKA)—cAMP response element binding protein (CREB) pathway [110]. In the in vitro assays CCKLR1 coupled to both the G_q_/G_11_ and G_i_/G_o_ signaling pathways [42] and the SK receptors of *T. castaneum* stimulated both the Ca^2+^ and cyclic AMP second messenger pathways [111]. Ligand-receptor interaction characteristics were modeled for the *T. castaneum* SKRs (TcSKR1 and TcSKR2) [112] and the structure–activity properties of different SKs were monitored in the cockroach hindgut contraction assay and it was found that the C-terminal heptapeptide DYGHMRFamide with sulfated tyrosine and amidation is critical for activity [95]. Using the flour beetle *T. castaneum* as a model, stable agonists and antagonists of SK were developed, injected and found effective on food intake [113].

As we shall see below, the two SKRs appear to be differentially distributed and contribute to distinct functions in invertebrates (in those few species that have been studied).

### Distribution of SK peptides and receptors in invertebrates

#### Peptide distribution

In general, the cellular SK peptide expression in the nervous system is conserved among studied insects, but the distribution in other arthropods has not been described in detail. Thus, it is hard to make wide comparisons even among the arthropod taxa. Also in the other invertebrates, data on the cellular SK distribution is scarce.

**Fig. 4:**
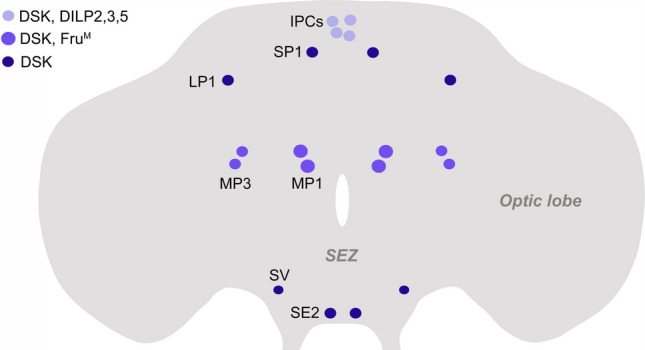
Distribution of cell bodies of neurons expressing DSK in the *Drosophila* brain. Four of the insulin-producing cells (IPCs) co-express DSK (the other IPCs are not shown). The MP1 and MP3 neurons express the male splice form of the transcription factor fruitless (Fru^M^). The designations of the DSK neurons are based on [114, 287, 288]

In insects SK expression is primarily seen in a small number of neurons and neurosecretory cells with cell bodies in the brain [114–118]. Generally, SK has not been found in neuronal cell bodies of the ventral nerve cord (VNC), or in enteroendocrine cells (EECs) of the intestine. One exception is the mosquito *Aedes aegypti* where SK immunoreactivity was detected in EECs of the midgut [119]. In the insect brain, the number of SK-producing neurons is small: for example, about 26 neurons have been detected in *R. prolixus* [116], 20–24 in *Drosophila* (Fig. 4) [23, 44, 114, 120, 121], and about 30 neurons in *P. americana* [117, 118]. In all these species the numbers of neurons include small sets of neurosecretory cells in the pars intercerebralis, with axon terminations in the corpora cardiaca and neurohemal areas on the anterior aorta, crop and foregut, suggesting roles of SKs as circulating hormones (or local modulators of endocrine cells or muscles). In *Drosophila,* it was shown that the DSK-expressing pars intercerebralis neurons are a subpopulation of the 14 insulin-producing cells (IPCs) [121, 122]. It cannot be excluded that the axon terminations of the DSK/DILP-containing IPCs on the crop release peptide that acts on crop muscle, as was shown for the peptide myosuppressin released from similar neurosecretory cells [123, 124]. All studied insect species appear to have one or two pairs of large SK-expressing neurons with cell bodies and extensive processes in the brain and axons descending throughout the VNC (see [23, 114–116, 118, 125]).

**Fig. 5:**
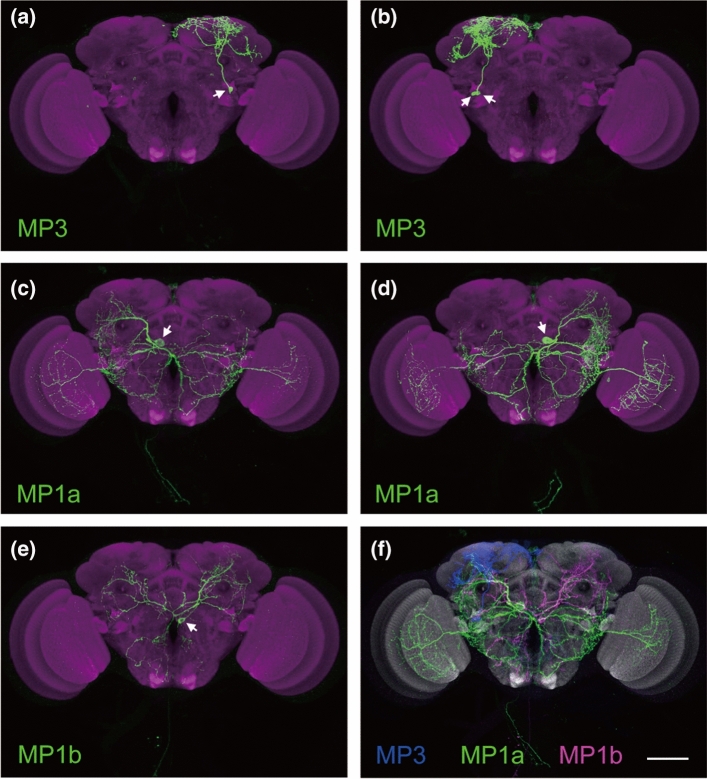
Single-neuron labeling of DSK-producing MP neurons. **a–f.** Stochastic labeling of single MP3 (**a**), two MP3 (**b**), single MP1a (**c** and **d**) and single MP1b (**e**) neurons. Two MP3, one MP1a and one MP1b neurons are registered in a standard brain (**f**). Scale bars, 50 μm. Note that the MP1/MP3 neurons do not innervate central complex, antennal lobes or mushroom bodies. Figure from Wu et al. [23], with permission

The available data on the cellular distribution of SK in insects suggests a rather unique pattern compared to many other brain neuropeptides (see [16]). In addition to being excluded from gut EECs, SK is expressed in a few neurons with wide arborizations that invade neuropils interspersed between the so-called structured neuropils (also known as glomerular or columnar/layered neuropils). One exception is the DSK processes invading the lobula of the optic lobe in *Drosophila,* which is a columnar neuropil. Thus, there are no reports of SK-expressing neuronal branches in the central complex, the mushroom bodies, the antennal lobes or the rest of the optic lobe of insects (see Fig. 5). Also, there are no reports on clock neurons expressing SK. The arborizations in the *Drosophila* lobula are diffuse and irregular and derived from the two MP1a neurons (each neuron with branches bilaterally in both lobulae) (Fig. 5c, d). In *Drosophila* and other insects, two pairs of brain neurons have axons that descend throughout the VNC [23, 44, 114, 115, 125]. In *Drosophila,* these are the MP1a and MP1b neurons [44].

There are other neuropeptides/peptide hormones in *Drosophila* that can be seen only in neurons with processes in non-structured neuropils. These are CAPA-PK, CCAP, DH44, GPB5, hugin-PK, DILPs, ITP, and LK (see [16], and furthermore they are not expressed in gut EECs [126]. Three of these peptides, DH44, hugin-PK, ITP, are however, also present in clock neurons [127, 128]. Interestingly the peptides listed above are known to regulate metabolism, feeding and/or water and ion homeostasis in adult flies (see [16]. In summary, it appears that in insects SK is not a brain–gut peptide, in contrast to many other peptides (see [1, 129, 130]). However, SK is likely to function both as a local neuromodulator and a circulating hormone. We will describe the detailed anatomy of the *Drosophila* DSK neurons in relation to their functions in a later section (Sect. 4).

In crustaceans, there is scarce information about cellular distribution of SK. In the shrimp *Penaeus monodon,* SK-immunolabeled neuronal cell bodies were seen only in the brain [88]. One pair of large cell bodies give rise to descending axons running throughout the VNC, similar to those in insects, and there are six to eight additional smaller brain neurons [88]. SK was detected in axon terminations of the neurohemal organ in the eyestalks designated the sinus gland [131] and in the neurohemal pericardial organ [132] of the crab *Cancer borealis*. CCK-type peptide was furthermore identified in neuronal processes in the stomatogastric nervous system (STN) of this crab [133, 134]. The STN is known to house neuronal circuits that regulate rhythmic movements of elements in the internal feeding apparatus such as the gastric mill (for chewing) and pylorus (for pumping and filtering of chewed food). The distribution of CCK-type peptide suggests that it may act both as a neuromodulator in local STN neurons, and as a circulating hormone to regulate rhythm-generating networks utilized during feeding and food processing [133, 134]. However, although several neuropeptides/peptide hormones have been investigated for their modulatory actions in the circuits of the STN, the function of CCK-type peptide remains to be tested.

In *C. elegans* the CCK-type neuropeptide NPL-12 is localized in one tail neuron with its cell body in the dorso-rectal ganglion, and was identified as the ring interneuron DVA [25]. This neuron connects to the SMB motoneuron that regulates head movements during feeding and ventral cord motoneurons controlling body wall muscles and thereby locomotion (see [27, 93]). The ventral cord motoneurons express the CCK receptor CKR2 and in presence of food the worms dwell and feed, whereas in absence of food they disperse under control of this circuit, which also includes sensory cells, as well as dopaminergic (PDE) and other peptidergic (AVK) neurons [27].

In the starfish *A. rubens* (Echinodermata) the CNS is organized in a radial fashion with a circumoral nerve ring and radial nerve cords. SK peptide was detected by in situ hybridization and immunohistochemistry to neuronal cell bodies and axonal processes in the so-called ectoneural and hyponeural regions of the CNS [32]. The neurons in the ectoneural region are predominantly sensory neurons and interneurons, whereas the hyponeural region houses motoneurons [32]. Furthermore these authors found SK neurons in the digestive system (esophagus, peristomial membrane, cardiac stomach, pyloric stomach, pyloric duct, pyloric caeca, intestine, and rectal caeca), tube feet and body wall. SK peptides were shown to trigger stomach retraction and inhibition of feeding in the starfish [32].

#### SK receptor distribution

The general distribution of SK receptors in major tissues has been assayed in some insects by PCR [100, 104, 105, 109]. There are no data for the two DSK receptors in tissues of *Drosophila* in FlyAtlas, probably due to low expression levels. The cellular distribution of SK receptors has to our knowledge only been investigated in *Drosophila* [23, 44, 45], the cockroach *P. americana* [118], and in the starfish *A. rubens* [32], but not in other invertebrates.

In *Drosophila*, different knock-in GAL4s into the gene loci of CCKLR-17D3 and CCKLR-17D1 were utilized for displaying the distribution of the DSK receptors [23, 44, 45]. The two receptors appear to be largely differentially expressed, but detailed analysis was not performed to screen for any colocalization or for detailed neuronal expression (but see below). Both receptors display widespread distributions in numerous neurons in the brain and VNC [23, 44, 45]. CCKLR-17D1 is expressed in neurons of the optic lobe, fan-shaped body of the central complex, SEZ and numerous protocerebral neurons [44]. CCKLR-17D3 is expressed in fan-shaped body of the central complex, mushroom bodies and in a smaller number of neurons scattered in the protocerebrum and SEZ [23, 44]. Furthermore, it was found that CCKLR-17D3 is expressed in subsets of gustatory receptor neurons (GRNs) in the proboscis and proleg tarsi that house the sweet sensing gustatory receptors Gr64f [45]. Recent work showed that CCKLR-17D1 is needed for modulation of aggression [44], whereas CCKLR-17D3 in suppressing sexual arousal and in sweet sensing [23, 45].

In the cockroach *P. americana* SK receptor protein was detected in octopaminergic dorsal unpaired median (DUM) neurons of the VNC and it was found that as a satiety signal SK depresses activity in these DUM neurons antagonistically to the orexigenic peptide AKH [118].

In the starfish *A. rubens,* the distribution of the single SK receptor was localized by immunocytochemistry to neurons in the CNS, tube feet, apical muscle, and digestive system [32]. Close apposition between SK peptide and SK receptor was seen in the ectoneural neuropil in the circumoral nerve ring and radial nerve cords. These authors furthermore found that some SK producing neurons also expressed the SK receptor.

### Functional roles of SK signaling in invertebrates

As seen in Table 1, SK signaling has been investigated in several species of insects, as well as in a few other invertebrates. Since *Drosophila* and the brown planthopper *Nilaparvata lugens* have been studied in some more detail we will describe DSK functions in these insects separately in the next section.

In several insect species injection of SK peptide and double stranded RNA for RNA-interference (RNAi) to knock down SK and SKR have been utilized to show that SK signaling decreases food intake (references in Table 1). DSK injections furthermore decrease secretion of digestive enzymes in a locust [135], but increased α-amylase secretion in the palm weevil *Rhynchophorus ferrugineus* [136]. Other effects of SK determined after injections or by in vitro assays are stimulation of gut muscle contractions [24], effects on fatty acid-, carbohydrate- and insulin-like peptide levels in beetles [100, 137]. In the ascidian *Styela clava* mammalian CCK8 peptide acts to stimulate gastric enzyme secretion [138] and in another ascidian *C. intestinalis* the distribution of SK receptor in the siphon and ovary suggests a role for CCK-type signaling in feeding and reproduction [102].

Could loss of SK signaling in some insects be correlated with altered physiology and/or behavior? Some papers suggest so. For instance, SK/SKR could not be found in the genomes of the peach aphid *Myzus persicae* and pea aphid *Acyrthosiphon pisum* [139]. Since SK/SKR plays an important role in feeding behavior and aphids excrete honeydew, which results in a substantial loss of energy, it was proposed that loss of the SK signaling system might lead to increased food intake to compensate for the energy loss [79]. Another suggestion is that a specific insect lifestyle could result in loss of SK signaling since parasitic wasps lack SK/SKR, whereas the social honeybee *A. mellifera* does not [78]. However, as mentioned above in an earlier section, lack of SK signaling could not be correlated with differences in feeding behaviors and other life history parameters or taxonomic relatedness of species in a study of 31 species of beetles 13 of which lack SKs [76]. Thus, it remains to be clarified whether a loss of SK signaling reflects behavior and physiology or if other peptidergic systems take over the role of SKs.

## Multiple functional roles of CCK-type signaling in *Drosophila*

Since DSK signaling in *Drosophila* has been quite well investigated at the cellular level, we provide a more detailed description here. We first outline the anatomy of the DSK neurons and the circuits they are part of. These circuits modulate aggression, courtship behavior, taste, foraging and feeding and seem to integrate multiple inputs from external and internal sensors. The studies reviewed here pertain to adult *Drosophila* males if nothing else is stated.

### DSK signaling in *Drosophila*

In the *Drosophila* brain there are 20 distinct DSK-expressing neurons (Fig. 4) and a small number of additional neurons that are less consistently seen with immunolabeling and Gal4-expression [23, 44, 45, 114]. No DSK-producing neuronal cell bodies were detected in the ventral nerve cord or intestine, although brain-derived axonal processes can be seen in the ventral nerve cord [44, 114]. The major types of DSK neurons that we will discuss here are a small subset of the IPCs in the pars intercerebralis [121, 122], and four pairs of median posterior neurons designated MP1 and MP3 [23, 44, 114]. The functions of the other DSK neurons are not yet known.

It is not clear how many of the 14 DILP-producing IPCs that co-express DSK, since the DSK expression is variable, but at least four cells can consistently be seen in adults [121]. The IPCs send axons to the retrocerebral complex (corpora cardiaca–corpora allata and hypocerebral ganglion) as well as the anterior aorta, foregut and crop and additionally have branches (presumed dendrites and/or peptide release sites) in pars intercerebralis, tritocerebrum and SEZ (see [149, 150]). DSK-immunolabeling can be seen in these brain processes, but the peptide distribution in the other sites has unfortunately not been examined in *Drosophila*. However, in the American cockroach, the corresponding neurons have SK expressing axon terminations in the corpora cardiaca–corpora allata [117].

The MP1 and MP3 neurons are of three distinct types shown in Fig. 5 [23, 44]. Each of the two pairs of MP3 neurons has wide ipsilateral arborizations dorsally in one brain hemisphere (Fig. 5a, b). The MP1a neurons are bilateral with branches in the lobula and ventrolateral brain neuropils (including subesophageal zone, SEZ) in both hemispheres (Fig. 5c, d), as well as axons descending into the VNC where they innervate the accessory mesothoracic neuropil. MP1b neurons are bilaterally supplying branches to the midbrain and SEZ (Fig. 5e) and have axons to the VNC. Together these DSK neurons innervate a substantial volume of the brain (Fig. 5f), but avoid the prominent centers such as the mushroom bodies, central complex and antennal lobes. Also the lamina, medulla and lobula plate of the optic lobe are devoid of DSK processes.

**Fig. 6:**
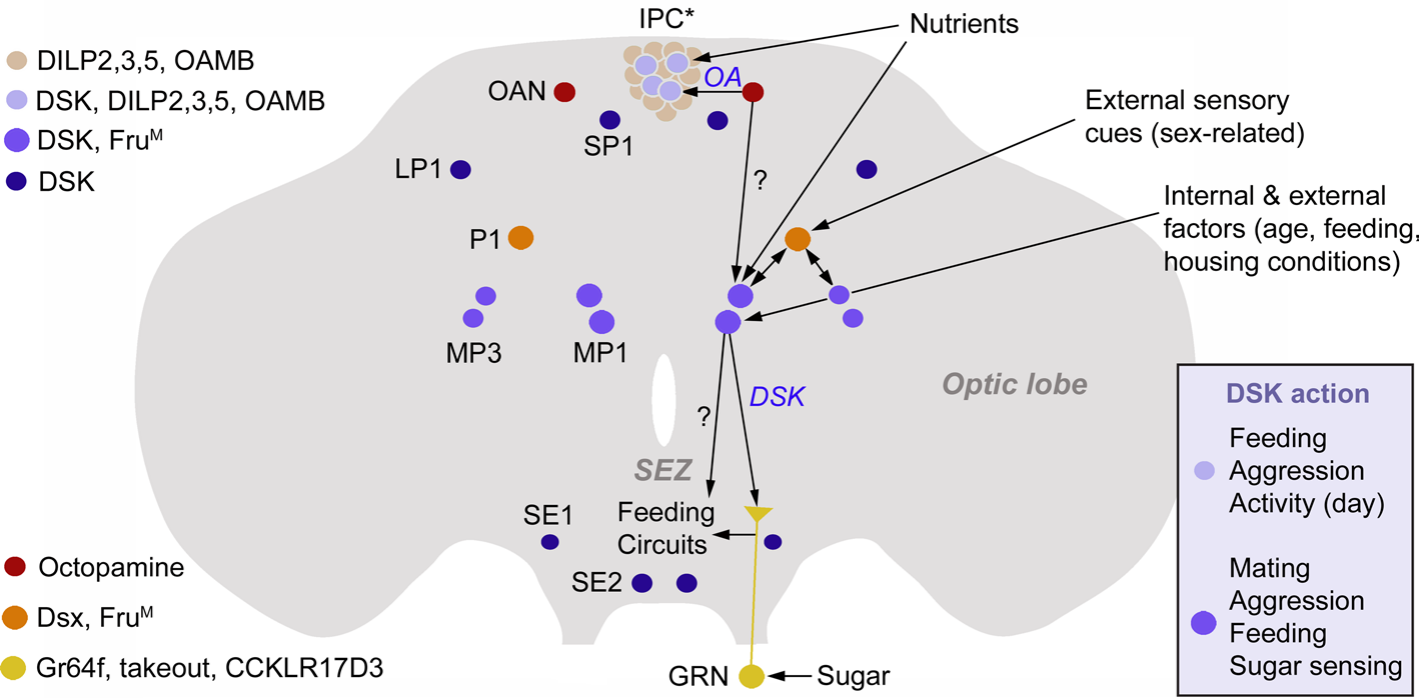
DSK-expressing neurons in the *Drosophila* brain and their interactions with other neurons in regulation of behavior and physiology. Of the DSK expressing neurons shown, only the MP1, MP3 and a subpopulation of insulin-producing cells (IPCs) in the pars intercerebralis have been specifically implicated in regulation of feeding/metabolism, aggression and mating behavior. Their functions are shown in the box with DSK action. The functional roles of the remaining (dark blue) neurons are not yet known. Other neurons shown are a pair of octopaminergic neurons (OAN), a pair (only a pair from a larger neuron cluster is shown) of fruitless^M^ (Fru^M^) and doublesex (Dsx) expressing neurons (P1) and a sweet-sensing gustatory receptor neuron (GRN) that expresses the gustatory receptor Gr64f, takeout and the DSK receptor CCKLR-17D3 (CCKLR). The OANs regulate activity in IPCs, probably including the ones expressing DSK, thereby regulating satiety (feeding), aggression and daily activity [140, 151]. It is not known if the OANs also interact with the MP1/MP3 neurons (indicated by ?). The MP1/MP3 neurons act on the P1 neurons to suppress male sexual behavior [23]. The P1 neurons also regulate activity in MP1/MP3 neurons to modulate male aggression, and MP1/MP3 neurons are postsynaptic to P1 neurons as shown by trans-tango [44]. Furthermore, the MP1/MP3 neurons receive inputs mediating internal and external cues about age, metabolic status, and housing conditions and thereby regulate mating, feeding and sugar sensing [23, 45]. After food intake, the MP1/MP3 neurons suppress activity in the Gr64f-expressing GRNs to diminish sugar sensitivity and thereby food search and feeding [45]. The GRNs signal to circuits that regulate motivation to feed (Feeding Circuits)

As seen in Fig. 6, the DSK-producing IPCs and the MP1/MP3 neurons are in male flies part of circuits regulating sugar sensing, feeding, daily activity, aggression and courtship behavior (females have not been specifically studied). Furthermore, this figure shows that the DSK expressing neurons are of different functional types (and molecular set-up). Thus, the IPCs express the octopamine receptor OAMB and are under influence of octopaminergic neurons [140, 151, 152], whereas the MP1/MP3 neurons express the male splice form of the transcription factor Fruitless (Fru^M^) and are connected reciprocally to the P1 neuron cluster [23, 44]. Both of these types of DSK neurons receive direct or indirect signals reporting nutritional status and the MP1/MP3 neurons are additionally influenced by other internal and external factors (age and housing conditions) [23, 45, 153]. It can be noted that the IPCs are furthermore regulated by a number of other neurotransmitters, neuropeptides and systemic inputs that will be discussed in a later section. The other DSK neuron types have not been specifically investigated. We shall get back to this figure in the next section in the context of functional DSK circuits.

As mentioned earlier, the distribution of the two DSK receptors CCKLR-17D1 and CCKLR-17D3 has been mapped in the *Drosophila* CNS by Gal4-driven fluorescence [23, 44]. The distribution has not been described at the cellular level though, except that a subset of the gustatory receptor neurons (GRNs) in the proboscis and proleg tarsi that also express the sugar receptor Gr64f have co-localized CCKLR-17D3 [45]. In male flies a subset of the P1 neurons also express CCKLR-17D3 [23]. Generally, the distribution of the two DSK receptors appear to match that of DSK producing neurons processes, but they can also be seen in processes in the fan-shaped body of the central complex (17D1 and 17D3), in the optic lobe (17D1 and 17D3), and in the mushroom body lobes (17D3) [23, 44, 45]. This reflects the Gal4-driven GFP expression in the receptor expressing neurons and not necessarily the distribution of receptor protein; the polarity of these neurons in terms of dendrites and axon terminations/output sites needs to be resolved.

#### Aggression and courtship behavior

**Fig. 7:**
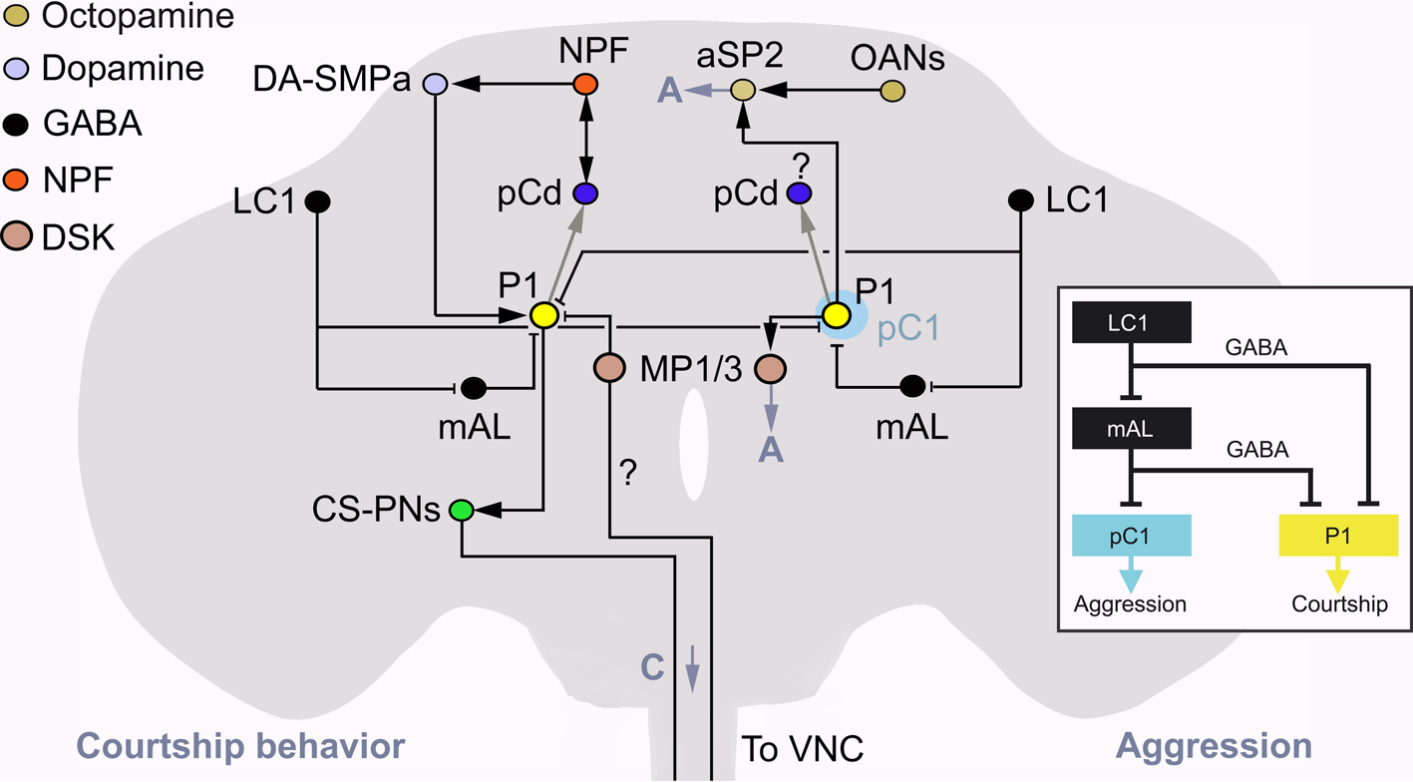
Neuronal circuits that regulate courtship behavior and aggression in *Drosophila*. In this simplified scheme, single neurons are shown even when multiple neurons are involved (e.g., P1, pC1, MP1/3 neurons). Circuits to the left regulate courtship behavior and those to the right aggression. Arrows show activation and stop bars inhibition. The gray arrows indicate indirect action via other neurons. The double arrows indicate a stimulatory recurrent circuit that involves pCD and NPF neurons that is known to sustain courtship motivation [167]. The neurotransmitters and neuropeptides utilized by some of the neurons are shown in the upper left corner. This scheme does not include the sensory inputs to P1 neurons and other neurons that mediate various signals from conspecific male and female flies. The P1 neurons are a subpopulation of the double-sex-expressing pC1 neurons (light blue circle), as shown to the right [157]. Approximately 20 P1 neurons (Fru^M^-expressing) are central in initiating courtship, whereas an unspecified number of P1/pC1 neurons trigger aggression [157, 161–163]. The DSK-expressing MP1/3 neurons are functionally associated with P1 neurons in both behaviors [23, 44]. Importantly, the Fru-expressing LC1 and mAL neurons use GABA to switch P1 (or P1/pC1) neuron activity between courtship and aggression [157]. Thus, LC1 neurons are shown here schematically as interconnecting P1 and P1/pC1 circuits reciprocally in the two behavior circuits. In the real fly, this circuit is present in each hemisphere as shown in the inset. There is an inhibitory recurrent signal from copulation-reporting neurons in the abdominal ganglion to brain NPF neurons (not shown here). In the VNC, another set of abdominal neurons utilize GABA, glutamate and corazonin to regulate copulation under modulation by dopamine. In aggression P1 neurons and octopaminergic neurons (OANs) converge on aSP2 neurons to promote aggression. Like in the courtship circuitry, pCd neurons ensure sustained aggression (A), but it is not known whether also here the NPF neurons are involved (indicated by ?). For further details, see the text. This figure is based on a figure in Lee and Wu [289] and is redrawn and updated with MP1/MP3 circuits

In male flies, the MP1 and MP3 neurons express *Fru*^*M*^ and receive inputs from the male-specific P1 neurons that also express *Fru*^*M*^ [23, 44] (Fig. 6). These DSK neurons are thus part of a circuit that regulates male-specific behavior. The P1 neurons constitute a heterologous set of about 20 neurons that express *Fru*^*M*^ and are known to integrate chemosensory and other cues from potential mates and control sexual arousal, or cues from other males and regulate aggression [154–160]. Thus, the P1 neurons weigh sensory inputs to control two opposing behaviors. Interestingly, the MP1/MP3 neurons play important roles in this circuitry by being postsynaptic to a subset of the P1 neurons and acting on *Fru*^*M*^ positive neurons that express the DSK receptor CCKL-17D3 for sexual arousal and CCKL-17D1 for aggression [23, 44]. In Fig. 7, we show a simplified scheme of the circuitry that regulates aggression and courtship behavior, including the MP1/MP3 neurons. These circuits are interconnected by GABAergic neurons that form a switch between courtship and aggression [157]. In Fig. 6, we show that P1 neurons and some DSK neurons receive inputs from the external and internal environment that provide cues for activation of specific circuits.

The P1 neurons are a subpopulation of the doublesex-expressing pC1 neurons [157]. About 20 P1 neurons receive various sensory cues from female flies and are central in initiating courtship, whereas an unspecified number of P1/pC1 neurons trigger aggression based on sensory cues from other males or non-conspecifics [157, 161–163]. The DSK-expressing MP1/MP3 neurons are functionally associated with P1 neurons in both behaviors [23, 44]. We shall start by describing the courtship circuitry (Fig. 7, left side). In courtship, the P1 neurons are triggered by sensory inputs and are modulated by dopamine from DA-SMPa neurons [164, 165], inhibited by DSK from MP1/3 neurons [23] and indirectly, via neuropeptide F receptor (NPFR) expressing DA-SMPa neurons, by NPF [166]. In support of the action of DSK from MP1/MP3 on P1 neurons, it was shown that P1 neurons express the receptor CCKLR-17D3 [23]. A stimulatory recurrent circuit including pCD and NPF neurons is known to sustain courtship motivation [167]. P1 neurons indirectly act on pCd neurons to extend duration of courtship singing [168]. A set of Fru-expressing LC1 and mAL neurons use GABA to switch P1 (or P1/pC1) neuron activity between courtship and aggression based on sensory cues from flies of either sex [157]. The P1 neurons act on descending neurons (known as P2b/plP10 neurons) to activate courtship singing via pacemaker circuits in the thoracic neuromeres of the VNC [169]. Via various inputs the MP1/MP3 neurons monitor internal and external signals such as metabolic status, housing conditions and ageing (Fig. 6) and based on the valence of these inputs they can inhibit the P1 neurons and thereby suppress courtship behavior [23]. The MP1/MP3 neurons have axons descending into the VNC [44, 114], but whether they are involved in regulating circuits controlling courtship in these ganglia is not known. After copulation there is an inhibitory recurrent signal from copulation reporting neurons in the abdominal ganglion to brain NPF neurons that ensure that the activation of P1 neurons cease [167]. Local circuits in the VNC comprise a set of abdominal neurons that utilize GABA, glutamate and corazonin to regulate copulation motor behavior under modulation by dopamine [170]. The MP1/MP3 neurons not only inhibit sexual arousal, they also suppress wakefulness and spontaneous walking, suggesting that DSK release has a general inhibitory effect on activity [23].

In virgin female flies, DSK also inhibits reproductive behavior via the CCKLR-17D3 receptor, by diminishing receptivity to courting males [23]. Female flies lack P1 neurons, but have doublesex-expressing neurons (e.g., PC1 and pCd) that are critical for female receptivity [171]. Labeling experiments suggest that the DSK neurons are presynaptic to the sex-specific doublesex neurons. Thus, both in males and females DSK neurons interact with the sex-dimporphic doublesex neurons to suppress reproductive behavior [23]. A recent study (preprint) also reported a role of DSK in female reproductive behavior [172]. These authors found that the DSK expressing MP1 neurons act on the CCKLR-17D3 receptor to regulate virgin female receptivity to mating males, as measured by copulation rate and copulation latency. Thus, the MP3 neurons appear to play no role in this behavior [172].

In male flies, P1 neurons and octopaminergic neurons (OANs) converge on aSP2 neurons to promote aggressive behavior [173] (Fig. 7). Like in the courtship circuitry, pCd neurons ensure sustained aggression [167], but it is not known whether also here the NPF neurons are involved in the circuitry, although NPF is involved in regulation of aggression [174]. As mentioned for courtship, the LC1-mAL circuit acts to switch P1 (or pC1) neuron activity between courtship and aggression. The MP1/MP3 neurons are postsynaptic to the P1 neurons and upon activation they promote aggression by acting on neurons expressing the DSK receptor CCKL-17D1 [44]. There are additional neurons that regulate aspects of aggressive behavior that utilize NPF, tachykinin and serotonin [174–176], but their relation to the MP1/MP3 circuits are not known (and they are not shown in Fig. 7).

It can be noted that also in mammals there is an association between circuits regulating aggression, reproduction and feeding, although not necessarily involving CCK. Such circuits are found in the hypothalamus and the neuroendocrine system, and it has been shown that neurons that are activated during aggression are inhibited during mating [177]. To some extent the functional analog of the hypothalamus in insects is the pars intercerebralis [178–180]. In *Drosophila*, this region houses several types of peptidergic neurosecretory cells, including the 14 IPCs that produce DILPs and DSK [121, 149, 180–182]. There is some evidence that the IPCs are involved in the regulation of male aggression [140, 141, 151, 183], and there seems to be to be a role of the co-localized DSK [140, 141], but the role of DSK in IPCs in courtship has not been tested.

#### Satiety and feeding in Drosophila and brown planthopper

Of the DSK expressing neurons, only the MP1, MP3 and a subpopulation of IPCs in the pars intercerebralis have been specifically implicated in regulation of satiety, feeding and metabolism [45, 121, 184] (Fig. 6). Knockdown of DSK in the IPCs or all DSK neurons, or inactivation of these neurons via expression of a hyperpolarizing ion channel, leads to flies that ingest more food [121]. Interestingly, it suffices to manipulate DSK in the IPCs alone to affect feeding. The same manipulations also affect the flies’ choice of food so that they are less discriminative against bitter food [121]. It was also found that knockdown of DSK in IPCs or all DSK neurons led to an increase in *dilp2, 3,* and *5* transcript levels in fed, but not in starved flies. Reversely, knockdown of *dilp2, 3,* and *5,* using a triple mutant, diminished *Dsk* levels in fed flies [121]. Thus, there seems to be an endocrine or autocrine feedback regulation of the two sets of neuropeptides. Another study also demonstrated that DSK knockdown in IPCs increased total amount of food ingested and number of feeding bouts [184]. That study found that octopamine increases feeding, but also *Dsk* transcription, suggesting a negative feedback between octopamine and DSK in regulation of feeding. Neither of these studies addressed the mechanisms by which DSK induces satiety and decreased food ingestion.

**Fig. 8:**
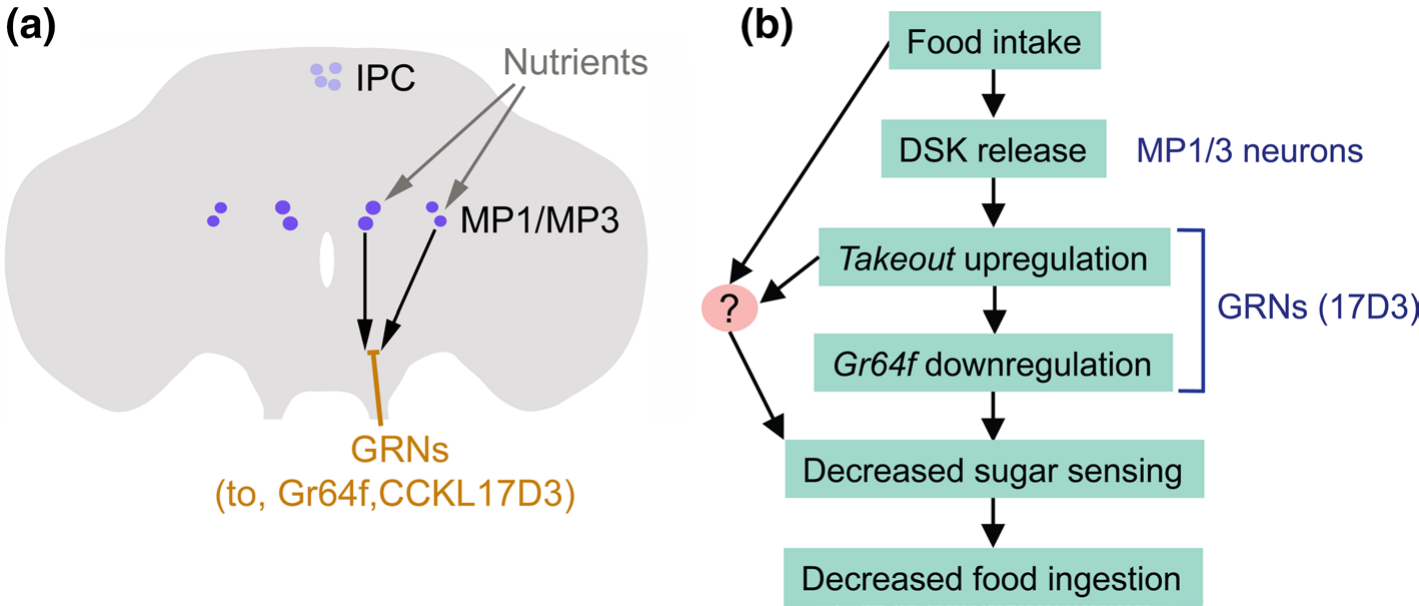
DSK regulates sugar sensitivity and feeding in *Drosophila*. (**a**) The MP1/MP3 neurons receive nutrient signals of unknown origin. Upon feeding DSK is released and inhibits responsiveness of afferent gustatory receptor neurons (GRNs) that express the DSK receptor CCKL17D3, as well as the sugar receptor Gr64f and the gene takeout (to). (**b**) Scheme depicting the action of MP1/MP3 neurons after food intake. It is likely that a parallel pathway acts on unknown neurons (indicated by ?) to decrease sugar sensing. Panel b is based on Guo et al. [45]

More recently, experiments on *Drosophila* and the brown planthopper *N. lugens* dissected the role of DSK signaling in satiety in some more detail [45]. This study focused on the MP1/MP3 neurons and their interactions with other neurons (see Fig. 8). Feeding upregulates *Dsk* transcription in the brain and more specifically induces elevated DSK immunolabeling in MP1/MP3 neurons, as well as increased spontaneous activity and calcium signaling in these neurons [45]. Thus, the MP1/MP3 neurons receive inputs from nutrient sensing neurons. Further analysis was guided by experiments in the planthopper. In this insect, an RNA-seq transcriptome analysis was performed after genetic downregulation of SK. Out of multiple genes with altered expression, a few genes of interest were found upregulated, namely those encoding sweet sensing gustatory receptor neurons (GRNs) and the *takeout* gene [45]. This is interesting since gustation is necessary for probing the palatability of food sources [185, 186] and *takeout* is important in feeding behavior of flies [187, 188]. Further experiments in *Drosophila* [45] showed that food ingestion downregulates the sweet gustatory receptor Gr64f (and starvation increases it). Optogenetic activation of GRNs that express Gr64f increases the flies’ motivation to feed. Furthermore, knockdown of *dsk* leads to an upregulation of Gr64f transcription and optogenetic activation of the DSK expressing MP1/MP3 neurons decreases the sugar sensitivity of gustatory neurons [45]. The DSK receptor CCKLR-17D3 could be found in a subpopulation of the Gr64f-expressing GRNs in proleg tarsi, proboscis and maxillary palps, and feeding dowregulates expression of this receptor in the appendages [45]. It was also found that silencing of the *Dsk* gene negatively regulates *takeout* expression and intriguingly knockdown of *takeout* leads to an upregulation of Gr64f expression. Thus, in summary, food intake leads to DSK release from MP1/MP3 neurons which upregulates *takeout* and downregulates Gr64f in CCKLR-17D3-expressing GRNs. This decreases the sugar sensing and thereby food ingestion is reduced (Fig. 8). In the planthopper similar mechanisms were revealed [45]. Thus, DSK signaling nutrient-dependently modulates the sensitivity of sweet-sensing GRNs both in *Drosophila* and the planthopper, suggesting a conserved peptidergic signaling pathway in these distantly related insects.

In *Drosophila*, it appears that the DSK-producing IPCs are not necessary for the interactions with sugar gustation and ensuing decreased feeding. This suggests that satiety signaling induced by DSK from the IPCs [121] is mechanistically distinct and further studies are required to determine whether it is by hormonal activity or maybe action on contractions of the crop, which is innervated by the IPCs. It is also important to note that the DSK/SK satiety signaling described by Guo et al. [45] is mechanistically very different from that shown for CCK in the mammalian gut—vagus nerve—hindbrain axis shown in Fig. 3. However, it might be that more similarities will be discovered in insects when compared to circuits in the brainstem and hypothalamus (see Sect. 2).

Since a subset of the IPCs produce DSK [121] it is possible that the mechanisms that are known to regulate production and release of DILPs affect the colocalized DSK in a similar way. This remains to be investigated. However, it was shown that *Dilp2, 3, 5* mutant flies display decreased DSK transcript levels and also that DSK-knockdown in DSK-neurons or in IPCs leads to increased *dilp* transcripts [121], suggesting feedbacks between these peptidergic systems. Thus, it is possible that the intrinsic nutrient sensitivity of IPCs and the neuropeptides and neurotransmitters that regulate IPC activity also affect DSK production and release. These signaling substances are listed in Table 2 and would be of interest to investigate in relation to DSK signaling.

**Table 2:** Neuropeptides, peptide hormones and neurotransmitters that regulate IPCs in adult *Drosophila*, some of which produce DSK

Peptide/SMN	Source	Receptor in IPCs	References
Allatostatin A		DAR2	[189]
CCHamide2	EECs	CCHa2-R	[190, 191]
DILP2,3,5	Autocrine feedback	dInR	[192]
DILP6	Fat body	dInR	[193]
DILP8	Tumors	Lgr3	[194]
Dopamine	Interneurons	Dopamine R1	[195]
DSK	Interneurons or autocrine	Not shown	[121]
GABA	Interneurons	GABA_B_R2	[196]
Leucokinin	Interneurons	LKR	[197, 198]
Limostatin	Corpora cardiaca	Pyrokinin receptor 1	[199]
NPF	EECs	NPFR	[200]
Octopamine	Interneurons	OAMB receptor	[151]
Pigment-dispersing factor	Clock neurons	PDFR	[201]
Serotonin	Interneurons	5-HT1A receptor	[202]
sNPF	Interneurons/LNCs	sNPFR1	[203]
Tachykinin	Interneurons	TkR99D (DTKR)^a^	[204]

^a^Another study found no TkR99D expression in IPCs and suggest that activation of IPCs is indirect via TkR99D expressing interneurons contacting IPCs [205]. In larval *Drosophila,* a pair of large TK expressing interneurons directly inhibit IPCs, but the specific receptor was not identified [206]

### Does DSK interact with other peptidergic and aminergic systems in *Drosophila*?

**Fig. 9:**
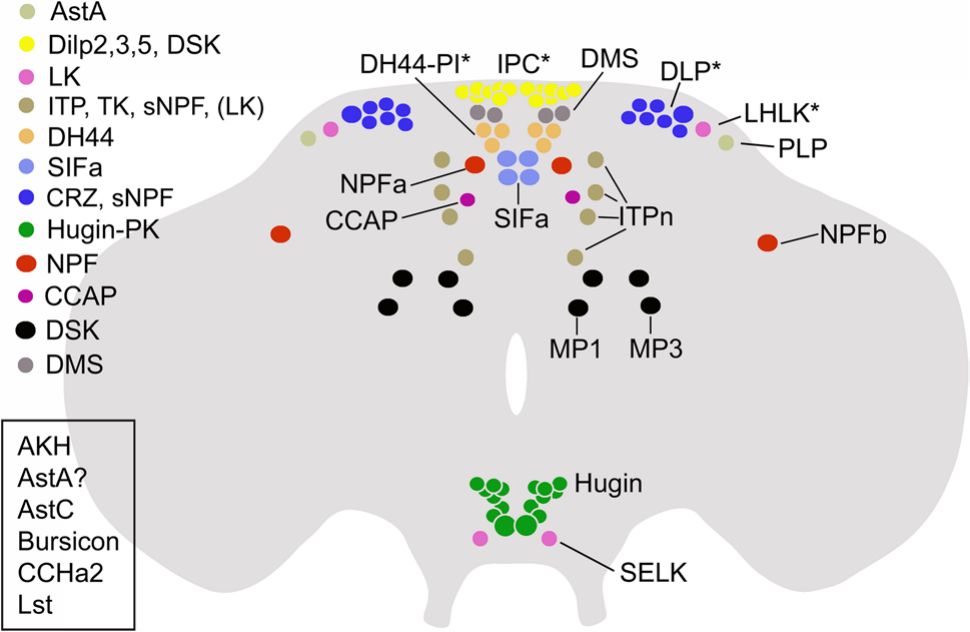
Peptidergic neuroendocrine systems in the *Drosophila* brain that regulate feeding and associated behaviors in addition to the DSK neurons. The figure shows the distribution of cell bodies of peptidergic neurons that have been implicated in feeding-related behavior. These are neurosecretory cells in MNC (IPC and DH44-PI) and LNC groups (ITPn and DLP) and interneurons located in distinct brain regions (MP1 and MP3, CCAP, LHLK, PLP, NPF, SIFa, Hugin and SELK); a few of the Hugin cells are neurosecretory cells. The neuron groups indicated with asterisks are cell autonomously nutrient sensing (only a subset of the DLPs), the MP1 and MP3 receive nutrient inputs, and the Hugin cells in the subesophageal zone receive gustatory inputs. The peptides released from these cells are shown in the legend in the upper left part of the figure. Note that also circuits associated with the mushroom bodies are linked to some of the peptidergic systems shown and are involved in regulation of food seeking and feeding [261]. There are also peptides derived from the intestine or corpora cardiaca that act on brain circuits to regulate feeding directly or indirectly (listed in the box in the bottom left of the figure). See text for further literature references. This figure was updated from Nässel and Zandawala [180]

There are several other neuropeptides involved in the regulation of feeding, aggression and courtship in *Drosophila*. They are listed in Tables 3, 4 and 5, and the peptide distribution in neurons involved in feeding is shown in Fig. 9. These peptides act at different levels of relevant circuits either as neuromodulators or as circulating hormones and their distribution in the brain is widespread with cell bodies in many different areas (Fig. 9). The details of the circuits involving these neurons are beyond the scope of this review and the reader is referred to the papers listed in the tables for further information.

**Table 3:** Neuropeptides that regulate aggression

Peptide	Neurons	References
DH44	DH44-R1 cells	[223]
DSK	IPCs, MP1/MP3	[44]
Natalisin	Interneurons	[224]
NPF	Interneurons	[174]
TK	Interneurons	[175]

**Table 4:** Neuropeptides that regulate mating and copulation

Peptide	Neurons	References
Corazonin	VNC neurons	[225, 226]
DH44	MNCs	[227]
DSK	MP1/MP3	[23]
MIP	Interneurons VNC (females)	[228]
Natalisin	Interneurons	[224]
NPF	Interneurons	[229–231]
PDF	Clock circuit	[229, 232]
Sex peptide	Sperm transfer	[232–235]
SIFa	Interneurons	[236, 237]

**Table 5:** Neuropeptides that regulate food search/feeding

Peptide	Neurons	References
AKH	Corpora cardiaca cells	[238, 239]
AstA	Interneurons, gut EECs	[189, 240–242]
CCHa2	Gut EECs, fat body	[190, 191]
CRZ	LNCs	[243]
DH44	MNCs and VNC neurons	[244–246]
DILPs	IPCs	[247–250]
DSK	IPCs, MP1/MP3	[45, 121]
Hugin	SEZ neurons	[251]
ITP	LNCs	[252]
LK	Brain neurons	[197, 253]
MIP	Brain neurons	[254, 255]
NPF	Interneurons	[256–262]
Sex peptide	Via sperm act in females	[263–266]
SIFamide	Interneurons	[267]
sNPF	OSNs-PNs, Interneurons MB circuits	[250, 257, 261, 268, 269]
TK	OSNs-PNs	[270]

Also monoamines like dopamine, octopamine, tyramine and serotonin play important roles in these behaviors. Octopaminergic neurons regulate courtship [207] or choice between courtship and aggression [208, 209], aggression [140, 210], appetite [211], and feeding behavior [184, 212, 213]. Tyramine is a satiety signal that in males supports courtship rather than feeding [214]. Dopaminergic neurons modulate courtship [164, 215], aggression [216], food search [217] and feeding behavior [218]. Serotonin modulates courtship [219, 220], aggression [176], and feeding behavior [221, 222]. In circuits that regulate aggression and courtship behavior, the MP1/MP2 neurons cooperate with neurons utilizing NPF, dopamine and octopamine as shown in Figs. 6, and 7, but in other cases it is not known whether the monoamines and other neuropeptides interact with DSK neurons and DSK signaling.

## Conclusions and future perspectives

In this review, we have discussed the evolutionary conservation of the structures of CCK-type peptides and their receptors and some of their functions in bilaterian invertebrates and vertebrates. The relatedness between invertebrate and mammalian CCK-type signaling components was suggested already many years ago [24, 26, 33, 42, 106], but now we can see that also several of the functional roles of CCK signaling appear to be evolutionarily conserved (see [32, 106, 271]). These include roles in satiety signaling and regulation of feeding, digestion, aggression and courtship behavior [18, 19, 23, 44–47, 62, 121, 135, 140, 272]. As will be discussed below, it is important to note that the “conserved functions” are only superficially similar and at a mechanistic level they differ in details and complexity between taxons. In contrast to the situation for mammals, there is not yet enough data in *Drosophila* or other invertebrates to reveal the complete circuits and pathways underlying DSK signaling in feeding, digestion, aggression and courtship behavior. In *Drosophila* some of this behavior regulation is by paracrine signaling in circuits within the CNS, and probably another part is by hormonal action via brain neurosecretory cells. In mammals, there are further layers of CCK signaling served by peripheral neurons (e.g., CCKR-expressing vagus nerve neurons, VANs) and gut EECs [18, 19, 46, 47]. These layers seem to be lacking in insects, but may be present in a simpler form in echinoderms [32]. It is, however, possible that DSK released from IPCs in *Drosophila* acts directly on the crop, which is supplied by axon terminations of the IPCs. This might regulate crop contractions and thus provide a satiety-induced effect similar to the gastric emptying triggered by the CCK-induced activity in the circuits of the vagus nerve afferents—brainstem efferents [18, 19, 46, 47].

It has been shown that CCK/SK act as local neuromodulators in different circuits of the CNS in both insects and mammals, but detailed comparisons of the circuitry remains to be performed [19, 23, 44, 46, 273, 274]. In mammals, CCK is co-expressed in various brain neurons with dopamine (DA), serotonin, GABA and other neuropeptides such as substance P, enkephalin, oxytocin and corticotropin-releasing hormone [46, 275, 276]; see also [277] for further co-expression patterns suggested from single cell transcriptomics data. Thus, CCK may function as a co-transmitter of dopamine, serotonin and GABA and as a co-modulator with other neuropeptides. Among invertebrates, we so far only know of the colocalization of DSK with DILPs in the IPCs of the *Drosophila* brain [121, 122], but systematic analysis has not been performed (however, see [278, 279]).

As noted above, the mechanisms by which CCK/SK induce satiety differ between insects and mammals. In mammals, gastric distension leads to CCK release from EECs of the gastrointestinal tract that trigger CCKR expressing afferent neurons (VANs) of the vagus nerve to signal to neurons in the brainstem, leading to a reduction in food intake [18, 46–48]. Concomitantly, second-order neurons in the nucleus of the solitary tract in the brainstem signal to several brain centers (including hypothalamus) that regulate feeding, reward and ingestion. In insects the CCK-mediated satiety signaling known so far is by means of circuits in the brain that affect carbohydrate sensing by gustatory neurons and thereby food intake, although DSK from the brain neurosecretory cells (IPCs) also seem to contribute to other satiety mechanisms [45, 121, 140]. In *C. elegans,* the CCK signaling acts to alter locomotion associated with feeding, rather than direct reduction of food intake [27] and in starfish CCK-type peptide acts on muscle to retract the stomach and thereby stop food intake [32]. There is a need to further investigate the insect circuits involving SK and feeding since we do not know how the metabolic state is conveyed to the SK neurons or the neuronal mechanisms for the reduced food ingestion upon satiety. The regulation of feeding is complex in mammals, and both the peripheral and central mechanisms involve numerous neurotransmitters and neuropeptides [18, 280]. Also in *Drosophila,* multiple brain circuits and peptidergic systems, as well as a few peptides released from EECs, have also been identified in regulation of feeding and metabolism (see Fig. 9 and Table 2). Due to this complexity, comparisons between mammals and insects regarding the complete circuitry are beyond the scope of this review. Also the DSK-associated circuits that regulate aggression and courtship behavior in *Drosophila* [23, 44] need further analysis and especially how the balance between the competing behaviors is accomplished.

Overall, the complexity of CCK/SK signaling varies depending on phylogenetic position of the organism and, thus, in mammals the range of pleiotropic actions is much wider than in insects. Yet we can show here that a relatively small number of DSK expressing neurons in the *Drosophila* brain have important roles in regulation of taste, satiety, feeding, activity, aggression and courtship. Most of these are interneurons that utilize DSKs in paracrine signaling in neuronal circuits within the CNS [23, 44, 45, 141], but a small set are neurosecretory cells (IPCs) that probably release DSKs and insulin-like peptides into the circulation for action on close or distant target tissues/organs, such as the crop, intestine and others [121]. In insects the hormonal action of SKs has not been explicitly verified by determination of peptides in the circulation. Some data, however, suggest hormonal action. Injection of SK regulates digestive enzyme activity and motility in the gut of insects, but the main part of the intestine is not innervated by SK producing neurons and no SK expressing EECs have been found [135, 136, 142, 147]. Thus, the endogenous source of SK acting on the intestine is likely to be the brain neurosecretory cells. To confirm this, SK release needs to be detected in the hemolymph and the distribution of DSK receptors in the periphery mapped to detect sites of action of SKs. Receptor expression data indicates low amounts of SKR1 and 2 transcript in heart and reproductive organs of *R. prolixus* [109], but data are lacking for other insects, including *Drosophila*.

One important finding is that DSK and specific small sets of DSK-producing neurons in *Drosophila* play a role in several behaviors, some of which are conflicting. These are the Fru^M^-expressing MP1/MP3 neurons that inhibit male courtship behavior, but stimulate aggression [23, 44]. The same neurons are also part of a satiety-inducing circuit that down-regulates sugar sensitivity and appetite in fed flies [45]. The circuits involved in regulation of aggression, courtship and satiety/feeding are each composed of multiple neurons that utilize a multitude of neurotransmitters and neuropeptides (Tables 3, 4 and 5). Nevertheless, the MP1/MP3 neurons appear to be involved in the switching between conflicting behaviors, probably by integrating different types of inputs that relay information about the external and internal environment. Other similar peptidergic brain systems are constituted for example by the four widely arborizing SIFamide-producing neurons that integrate sexual behavior, feeding and sleep by interactions with multiple brain and VNC circuits [237, 267, 281, 282] and Hugin neurons that integrate homeostatic sleep signals and the circadian clock, and relays locomotor activity output in adults [283, 284]. In larvae, Hugin neurons receive gustatory inputs and form a hub between feeding and locomotion [251, 285], a function that is not yet explored in adult flies.

In conclusion, we still have a long way to go to fully understand the fascinating roles of CCK/SK and their receptors in physiology and behavior of invertebrates. This peptidergic system is also interesting from the evolutionary point of view, since it illustrates how CCK-type signaling can induce specific states such as satiety in mechanistically distinct ways in insects and mammals.

## Supplementary Information

Below is the link to the electronic supplementary material.

Supplementary file1 (PDF 101 KB)

Supplementary file2 (PDF 1707 KB)

## Data Availability

This article is a literature review and does not contain any new data. Source data files for Figs. 1 and 2 are in Supplementary material.

## Abbreviations

AKH: Adipokinetic hormone
AstA: Allatostatin A
CAPA-PK: Capability pyrokinin
CCAP: Crustacean cardioactive peptide
CCHa2: CCHamide-2
CCK: Cholecystokinin
CRZ: Corazonin
DH44: Diuretic hormone 44
DILP: *Drosophila* Insulin-like peptide
DSK: *Drosophila* Sulfakinin
GABA: Gamma aminobutyric acid
GPB5: Glycoprotein B5
Hugin-PK: Hugin-derived pyrokinin
ILP: Insulin-like peptide
ITP: Ion transport peptide
LK: Leucokinin
MIP: Myoinhibitory peptide
NPF: Neuropeptide F
PDF: Pigment-dispersing factor
SIFa: SIFamide
SK: Sulfakinin
sNPF: Short neuropeptide F
TK: Tachykinin
