# Supplementary material for: Cholecystokinin/sulfakinin peptide signaling: conserved roles at the intersection between feeding, mating and aggression
Source: Cell Mol Life Sci. 2022 Mar 14;79(3):188. doi: 10.1007/s00018-022-04214-4 (PMC8921109; doi:10.1007/s00018-022-04214-4)
Supplement: Supplementary file 1 — Supplementary file1 (PDF 101 KB) [file 18_2022_4214_MOESM1_ESM.pdf]

Supplementary Data file 1 (Nässel and Wu)

Figure 1 – source data. Accession numbers for precursors of the neuropeptides shown in the sequence alignment in Figure 1.

Precursor/Peptide name	Species name	Accession number or PubMed reference ID
CCK	*Aplysia californica*	XP_005096263.1
CCK	*Asterias rubens*	ALJ99958
NP12	*Caenorhabditis elegans*	O01970
Cionin	*Ciona intestinalis*	P16240
SK	*Crassostrea virginica*	XP_022326792.1
SK	*Drosophila melanogaster*	P09040
CCK	*Homo sapiens*	P06307
Gastrin	*Homo sapiens*	P01350
SK	*Urechis unicinctus*	QUP52013.1
SK	*Capitella teleta*	ELT92762.1
SK	*Zophobas atratus*	Marciniak et al., 2011
SK	*Tribolium castaneum*	EFA04708.1
SK	*Periplaneta americana*	ALG35946.1
SK	*Anopheles gambiae*	AAR03495.1
SK	*Blattella germanica*	P85555.1
SK	*Leucophaea maderae*	P67802.1, P04428.1
SK	*Delia radicum*	Audsley et al., 2011
SK	*Apis mellifera*	XP_006557714.2
SK	*Chrysis viridula*	GATY02009227.1
SK	*Rhodnius prolixus*	ACT35306.1
SK	*Nilaparvata lugens*	XP_039279256.1
SK	*Gryllus bimaculatus*	CAL48349.1

SK	*Locusta migratoria*	P47733.1
SK	*Bombyx mori*	BAG49564.1
SK	*Phoronis australis*	Daniel Thiel et al., 2021
SK	*Lingula anatina*	Daniel Thiel et al., 2021
SK	*Notospermus geniculatus*	Daniel Thiel et al., 2021
