# Supplementary material for: Cholecystokinin/sulfakinin peptide signaling: conserved roles at the intersection between feeding, mating and aggression
Source: Cell Mol Life Sci. 2022 Mar 14;79(3):188. doi: 10.1007/s00018-022-04214-4 (PMC8921109; doi:10.1007/s00018-022-04214-4)
Supplement: Supplementary file 2 — Supplementary file2 (PDF 1707 KB) [file 18_2022_4214_MOESM2_ESM.pdf]

Supplementary Data file 1 (Nässel and Wu)

Figure 1 – source data. Accession numbers for precursors of the neuropeptides shown in Figure 2.

>sp|P06307.1| [Homo sapiens] CCK8
MNSGVCLCVLMAVLAAGALTQPVPPADPAGSGLQRAEEAPRRQLRVSQRTDG
ESRAHLGALLARYIQQARKAPSGRMSIVKNLQNLDPSHRISDRDYMGWMDF
GRRSAEEYEYPS

>NP_000796.1 [Homo sapiens] gastrin14
MQRLCVYVLIFALALAAFSEASWKPRSQQPDAPLGTGANRDLELPWLEQQGP
ASHHRRQLGPQGPPHLVADPSKKQGPWLEEEEEAYGWMDFGRRSAEDEN

>sp|P16240.2| [Ciona intestinalis] Cionin
MGSNIVIYFSIIVIVTLNVNGVPASDLFKSVSQYHIPRSKVINKETVTKPLQFQR
AICRLLQKLGEETFARLSQSELEAKQLDLIKTCYQANSFGDNENQGHMQRMD
RNYYGWMDFGKRAIEDVDYEY

> (Daniel Thiel et al., 2021) [Phoronis australis] sulfakinin
MKNLIRSVLPLISICQLVHVANSAPFFTKVPQHSIMGFVDILKDFSSRHPHTKEL
DQRPEQPQEMGPKQGEYGAKDMLYPILVSSKAEERFEPLSQRTLFGQKLSKPS
EANQDVENLYDIPNDKRDYGIGGGRFGKRKSPTKKTPRSRYFYGGGRFGRSV
ANFDHVDPETEEAFD

> (Daniel Thiel et al., 2021) [Lingula anatina] sulfakinin
MKSVFFVCIFFTTIVINVDCAPWQIGNRLRTSHISQAMHKLQGSLHRIFYLGRK
KPNSGQYFTNSFSTLKGGEPITNIYPMKGYIQVDQTKPVAASMQLKAFNGMD
SPTTGKQEKRAWDDYGLGGGRYGKREWKDGTSGLTLTQDATKHSNGNTNSK
RSDIYWLAGGRFGRDVSHVTDHVDPENDDDIEN

> (Daniel Thiel et al., 2021) [Notospermus geniculatus] sulfakinin
MNQALIIGVALFVVCASALPHKRTENAAGAKWSPSHTNHYQPEQFEKVFNAL
KGKDTDRYDGLLALVAAAGDEQLAEKRQVDMDYGWSGGRFGKRSVDNND
HIF

>EKC26412.1 [Crassostrea gigas] sulfakinin
MKNSLEHLLISSVLVLCLLFTLTKGNSHAQNSIIQLSKLFSNLQDIHDAKQTQE
ENTKDQVRSDTRKQRGEIGLILALGAPVHVESLQHNDKDTQTDTDKSAWDV
DFEDDQQDVEKRQGAWDYDYGLGGGRFGKRFDYNFGGGRWGRDVDHVKQ
GQAK

>XP_005096263.1 [Aplysia californica] sulfakinin
MEPQLLSVVILVLCACAASALPTNSRREDLSHLVTLLGKLKNIERARQNSEGS

AQAHAQEAWEGPRVSSFPETEVTADVIPDEVSAAKGYLGQRPATGVVNKRQG
AWSYDYGLGGGRFGKRSYGDYGIGGGRFGRDVDHVDLSDATENEITS

>NP_490908.1 [Caenorhabditis elegans] sulfakinin
MLRHHSCALLMLILVFVEVFATQSPTFDRQDRDYRPLQFGKRDGYRPLQFGK
RDYRPLQFGKRSSGSSGPVVLEPIWEWQ

>NP_524845.2 [Drosophila melanogaster] sulfakinin
MGPRSCTHFATLFMPLWALAFCFLVVLPIPAQTTSLQNAKDDRRLQELESKIGG
EIDQPIANLVGPSFSLFGDRRNQKTMSFGRRVPLISRPIIPIELDLLMDNDDERT
KAKRFDDYGHMRFGKRGGDDQFDDYGHMRFGR

>XP_039279256.1 [Nilaparvata lugens] sulfakinin
MGCSTMTAVLLTVSVFLLLQHQVGVLANGAAASELMTSSSNDLVTANRGQT
GRRRASLPVILSQARPNSKGGQLIRARLTPLEPLISDLLIDDVDDMMEIGKRQS
DDYGHMRFGKRGEADDKFDDYGHMRFGRDHV

>BAG49564.1 [Bombyx mori] sulfakinin
MRIAAVMLLAVSVAVTFCVCCCDGANLRRVQPDDDEDFRPHPLYRDYGLIRSR
VIRGDDTFDDYGHLRFGRSDD
